# Immune Cell Modulation of the Extracellular Matrix Contributes to the Pathogenesis of Pancreatic Cancer

**DOI:** 10.3390/biom11060901

**Published:** 2021-06-17

**Authors:** Ramiz S. Ahmad, Timothy D. Eubank, Slawomir Lukomski, Brian A. Boone

**Affiliations:** 1 Department of Surgery, West Virginia University, Morgantown, WV 26506, USA; ramiz.ahmad@hsc.wvu.edu; 2 Department of Microbiology, Immunology and Cell Biology, West Virginia University, Morgantown, WV 26506, USA; tdeubank@hsc.wvu.edu (T.D.E.); slukomski@hsc.wvu.edu (S.L.); 3 West Virginia University Cancer Institute, West Virginia University, Morgantown, WV 26506, USA

**Keywords:** pancreatic ductal adenocarcinoma, extracellular matrix, fibrosis, immune cell modulation, neutrophils, neutrophil extracellular trap, macrophages

## Abstract

Pancreatic ductal adenocarcinoma (PDAC) is a highly lethal malignancy with a five-year survival rate of only 9%. PDAC is characterized by a dense, fibrotic stroma composed of extracellular matrix (ECM) proteins. This desmoplastic stroma is a hallmark of PDAC, representing a significant physical barrier that is immunosuppressive and obstructs penetration of cytotoxic chemotherapy agents into the tumor microenvironment (TME). Additionally, dense ECM promotes hypoxia, making tumor cells refractive to radiation therapy and alters their metabolism, thereby supporting proliferation and survival. In this review, we outline the significant contribution of fibrosis to the pathogenesis of pancreatic cancer, with a focus on the cross talk between immune cells and pancreatic stellate cells that contribute to ECM deposition. We emphasize the cellular mechanisms by which neutrophils and macrophages, specifically, modulate the ECM in favor of PDAC-progression. Furthermore, we investigate how activated stellate cells and ECM influence immune cells and promote immunosuppression in PDAC. Finally, we summarize therapeutic strategies that target the stroma and hinder immune cell promotion of fibrogenesis, which have unfortunately led to mixed results. An enhanced understanding of the complex interactions between the pancreatic tumor ECM and immune cells may uncover novel treatment strategies that are desperately needed for this devastating disease.

## 1. Introduction

**Figure 1:**
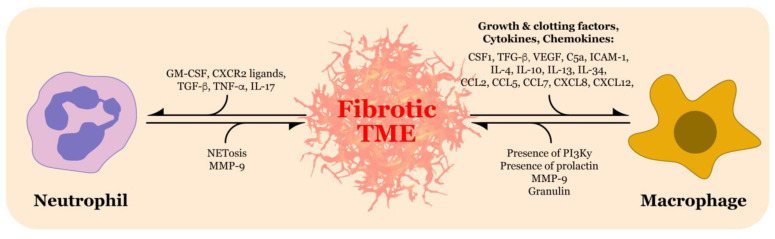
Crosstalk between the fibrotic tumor microenvironment (TME) and neutrophils and macrophages in pancreatic ductal adenocarcinoma. The fibrotic TME releases a variety of chemical mediators that recruit neutrophils and macrophages into the TME. In turn, neutrophils and macrophages possess characteristics and/or release their own factors that increase TME fibrosis. CSF1, colony stimulating factor 1; TGF-β, transforming growth factor-β; VEGF, vascular endothelial growth factor-A; C5a, complement component C5a; ICAM-1, intercellular adhesion molecule-1; IL, interleukin; CCL, chemokine (C-C motif) ligand; CXCL, chemokine (C-X-C motif) ligand; PI3Ky, phosphatidylinositol 3-kinase gamma; MMP-9, matrix metalloproteinase 9; GM-CSF, granulocyte-macrophage colony-stimulating factor; TNF-α, tumor necrosis factor-alpha; NETosis, neutrophil extracellular trap release.

Pancreatic ductal adenocarcinoma (PDAC) is one of the most lethal malignancies of the gastrointestinal system, with a five-year survival rate of only 9% [1]. It is currently the seventh-leading cause of deaths among cancers worldwide [2], and the incidence continues to rise [1,3]. At the time of pancreatic cancer diagnosis, up to 80% of patients present with metastatic or unresectable disease [4]. Pancreatic cancer is immensely difficult to treat, largely due to the dense, fibrotic stroma that dominates much of the tumor microenvironment (TME) [5]. A significant portion of the stroma is composed of extracellular matrix (ECM) proteins deposited through a desmoplastic reaction [6]. Desmoplasia is a fibro-inflammatory process of the stroma that consists of immune cells, proliferative fibroblasts, and abundant deposition of ECM proteins such as collagens and fibronectin [7,8,9,10]. While several therapeutic strategies for PDAC exist, the fibrotic stroma is a significant barrier to drug efficacy. Recent investigations have implicated immune cells such as neutrophils and macrophages in their contributions to the PDAC fibrotic stroma (Figure 1). In this review, we outline how the PDAC TME is established and the significant contribution of fibrosis to the pathogenesis, therapeutic resistance, metabolic adaptation, and immunosuppressive nature of PDAC. Moreover, we summarize the recent literature available on neutrophils and macrophages promoting PDAC fibrosis. Lastly, because of the therapeutic challenges presented by desmoplasia, we discuss current therapeutic strategies that target these immune cells with the aim of reducing PDAC fibrosis. 

## 2. The Pancreatic Adenocarcinoma Tumor Microenvironment

The pancreatic TME consists of cellular and acellular components including ductal epithelial cells, fibroblasts, myofibroblasts, activated pancreatic stellate cells (PSCs), and a host of immune cells including regulatory T cells, myeloid-derived suppressor cells, tumor associated macrophages (TAMs), and tumor associated neutrophils (TANs) [11,12].

Quiescent fibroblasts are cells that comprise most of the stroma in various tissues. They are intimately involved in ECM modulation by secreting numerous ECM proteins such as collagens, elastin, and fibronectin [13,14]. Fibroblasts are typically recruited to an area of tissue insult by transforming growth factor-β (TGF-β), platelet-derived growth factor (PDGF), and fibroblast growth factor 2 (FGF2). After their recruitment, the fibroblasts are activated and promote the wound healing response through both cytoskeletal and ECM remodeling. After resolution of the injury, fibroblast activation is reversible through apoptosis. However, if the signals associated with tissue damage such as TGF-β, PDGF, and FGF2 are incessant, as is the situation in malignancy, the activated fibroblasts become hyper-proliferative and can become cancer-associated fibroblasts (CAFs) [15]. 

The PDAC stroma is imbued with a heterogeneous and plastic population of CAFs [6,11,16]. Fibroblasts inside the tumor mass differentiate into CAFs when exposed TGF-β, produced by PDAC cells, stromal cells, and TAMs [17,18]. The conversion of fibroblasts into CAFs is a positive feedback loop, as the formation of CAFs mechanically releases more TGF-β from its binding protein, latency-associated peptide (LAP) [19]. The CAFs have an active role in the TME, where they enable tumorigenic functions through the release of several pro-inflammatory cytokines, such as TGF-β, granulocyte-macrophage colony-stimulating factor (GM-CSF), colony stimulating factor 1 (CSF-1), and CCL2 [16,20,21]. Moreover, CAFs secrete multiple growth factors such as vascular endothelial growth factor-A (VEGF-A), hepatocyte growth factor (HGF), and platelet-derived growth factor (PDGF). These growth factors facilitate recruitment of immune cells and endothelial cells into the TME [19].

An important fibroblast population known as pancreatic stellate cells (PSC) are quiescent, star-shaped cells that reside in the basolateral portions of pancreatic acinar cells. Notable intracellular characteristics of PSCs include a large nucleus, limited mitochondria, and copious amounts of vitamin A- and albumin-containing fat droplets [22]. The role of quiescent PSCs has not yet been fully elucidated, however, it is thought that they are involved in structural support of the basement membrane by providing scaffolding [23]. During tumorigenesis, PSCs can become activated by the stimulating factors such as TGF-β, Interleukin-10 (IL-10), and PDGF that are released from PDAC cells and stromal cells [24]. Once activated, PSCs represent the most common subpopulation of CAFs [6,25]. In contrast to their quiescent counterparts, activated PSCs do not possess fat droplets. The mechanism by which fat droplets disappear or the impact of their absence on PDAC progression has not yet been answered [23,26]. The physiological hallmark of PSC activation is the expression of α-smooth muscle actin (α-SMA), a type of cytoskeletal protein [27]. Activated PSCs are a key contributor to the PDAC fibrotic stroma as they increasingly release ECM proteins such as collagen, periostin, fibronectin, matrix metalloproteinases (MMPs), and tissue inhibitors of matrix metalloproteinases (TIMPs) [25,28,29]. There have been conflicting results on the association of MMP type with patient survival, but increased MMP-7 has more consistently correlated with poor patient survival [30]. 

A recent proteomic analysis of the ECM in PDAC progression found that elevated levels of fibronectin and periostin were significantly associated with worse patient outcomes [31]. Increased deposition of collagen I and collagen IV correlate with reduced patient survival, whereas collagen III levels do not have a statistically significant association with patient survival [32]. Further, it was found that high circulating levels of collagen IV after PDAC resection correlated with reduced patient survival [33]. Interestingly, high alignment of collagens in PDAC tumors is associated with poor patient survival and correlates to stromal activation [34]. 

## 3. Contributions of Fibrosis in the TME to Pathogenesis of Pancreatic Cancer

**Figure 2:**
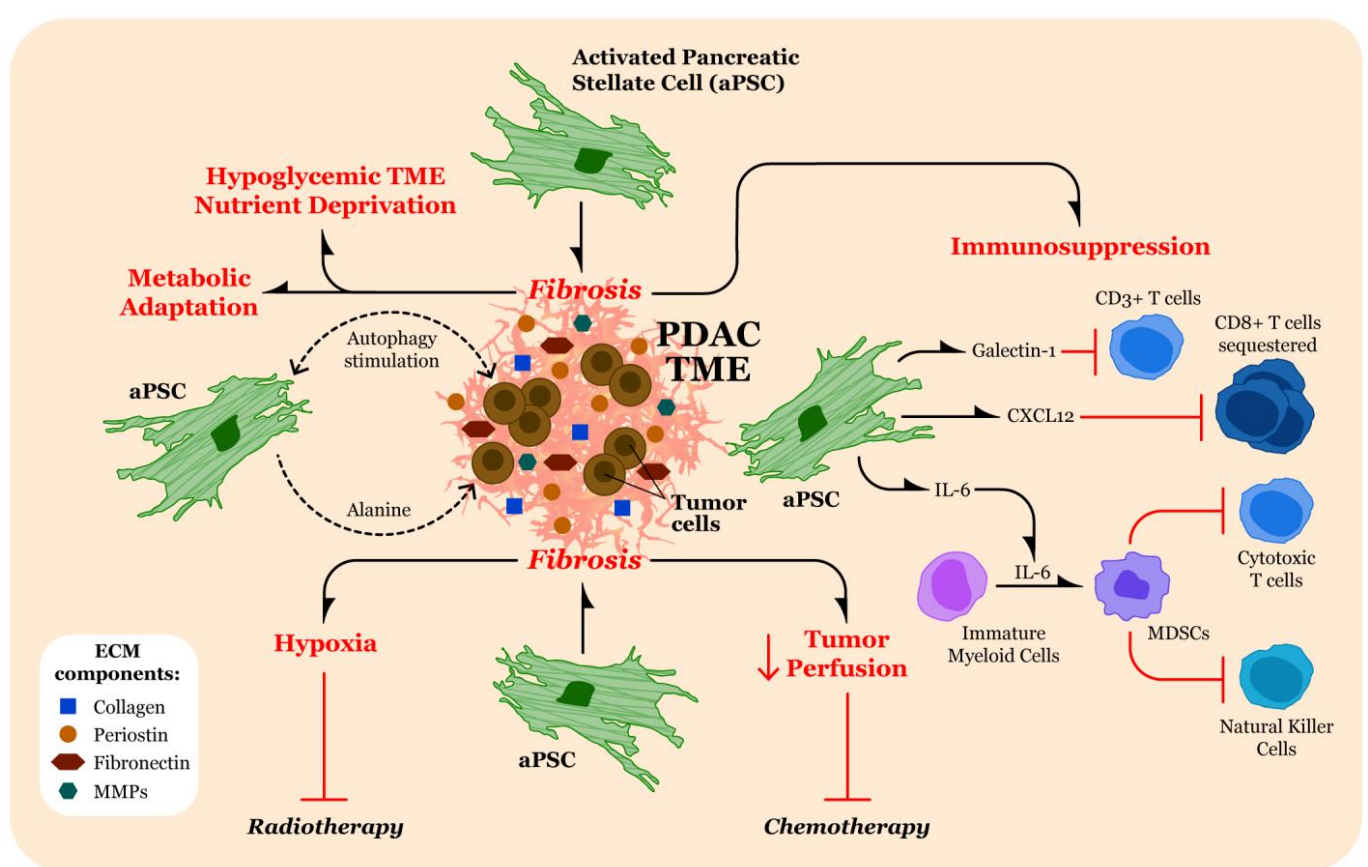
Contribution of extracellular matrix (ECM)/fibrosis to pancreatic ductal adenocarcinoma (PDAC) pathogenesis. Activated pancreatic stellate cells (aPSC) release significant quantities of ECM components, including collagen, periostin, fibronectin, and matrix metalloproteinases (MMPs) into the PDAC tumor microenvironment (TME) that contribute to fibrosis. The fibrotic PDAC TME results in several pathogenic effects (noted in red text). The abundance of fibrotic material in the PDAC TME can result in hypoxia and decreased tumor perfusion, which inhibit the therapeutic effects of radiotherapy and chemotherapy, respectively. Moreover, fibrosis can lead to hypoglycemia and nutrient deprivation in the TME. In response to nutrient deprivation, PDAC tumor cells metabolically adapt by stimulating autophagy in aPSCs, leading to release of alanine from aPSCs, which is then used for fuel by the PDAC tumor cells. The aPSCs may also stimulate autophagy in PDAC tumor cells. Immunosuppression in the TME is established in part by the release of galectin-1 and CXCL12 by aPSCs, inhibiting CD3^+^ T cells and sequestering CD8^+^ T cells, respectively. Additionally, release of interleukin-6 (IL-6) by aPSCs results in conversion of immature myeloid cells to myeloid-derived suppressor cells (MDSCs), which then inhibit infiltration by cytotoxic T cells and natural killer cells.

There are multiple mechanisms through which the characteristic PDAC TME facilitates PDAC progression by enhancing tumor growth and promoting metastases (Figure 2).

### 3.1. Therapeutic Resistance through Limiting Penetration of Cytotoxic Agents

The overabundance and imbalance of released ECM proteins establishes a high interstitial pressure environment resulting in decreased perfusion of the tumor. This high pressure and diminished perfusion prevent infiltration of cytotoxic agents into the TME [35,36]. Several treatment strategies that target the stroma, outlined below, have been designed to enhance penetration of chemotherapy to the tumor, thereby increasing efficacy and treatment response. 

### 3.2. Promotion of Hypoxia in the TME

Deposition of ECM proteins amplifies tissue tension and intra-tumoral pressure. These effects disrupt local blood circulation and oxygen diffusion in pancreatic tissue, leading to hypoxia [37,38]. In response to the hypoxic environment, hypoxia-inducible transcription factors (HIFs) are stabilized. The HIF-α/ARNT heterodimer, along with transcriptional coactivators, bind to hypoxia response elements (HREs) in gene promoters leading to transcription of genes in PDAC cells that facilitate glycolysis, tumorigenesis, and metastasis [39]. PSC exposure to HIF-α also increases expression of type I collagen, fibronectin, and periostin, thereby accentuating hypoxia secondary to ECM deposition and increased TME fibrosis [24,28,39,40].

The hypoxic TME also poses a significant challenge for radiotherapy interventions. Typically, radiation absorbed by the tissue requires oxygen to produce reactive oxygen species (ROS) that cause DNA damage to cancer cells, thereby shrinking the tumor. In the setting of hypoxia (under 10 mmHg), the efficacy of radiotherapy decreases and requires significantly increased dosage to reach the desired therapeutic effect [41]. Different strategies designed to alleviate hypoxia in the TME and improve radiotherapy penetration such as radiosensitizer drugs have been proposed [42,43] but will need to be tested against PDAC tumors. 

### 3.3. Altering Tumor Cell Metabolism

The reduction in perfusion associated with PDAC fibrosis also leads to nutrient deprivation of the tumor. To overcome this obstacle, PDAC cells maintain adequate nutrition by altering their metabolism to support tumor growth. The vast majority of PDAC cells possess the oncogenic KRAS mutation, enabling them to utilize glutamine as a nutritional source for cancer growth [44]. Aside from providing nutrition, glutamine is also used to promote hyaluronan production via the hexosamine biosynthesis pathway. A study done by Sherma et al. demonstrated that a small molecule glutamine analog (6-diazo-5-oxo-l-norleucine (DON)) was able to reduce the ECM deposition surrounding PDAC cells by inhibiting hexosamine biosynthesis [45]. KRAS mutant PDAC cells are also able to sustain themselves through macro-autophagy, a metabolic cell-survival process that relies on recycling of damaged organelles and proteins. Oncogenic KRAS positively regulates PDAC autophagy by promoting expression of vacuole membrane protein 1 (VMP1), a critical element of autophagosome formation [46,47]. There is also evidence that PDAC cells and activated PSCs engage in metabolic cross talk. Under hypoglycemic conditions, activated PSCs, induced into autophagy by PDAC cells, release alanine in the TME. The alanine is then internalized by PDAC cells and converted to pyruvate, which substitutes for glucose and glutamine in the tricarboxylic acid (TCA) cycle to maintain ATP generation [48,49]. 

### 3.4. Immunomodulation

In addition to secreting ECM proteins that provide a physical barrier to cytotoxic immune cells, activated PSCs release a variety of immunomodulatory factors that drive the PDAC TME into an immunosuppressive environment. One example is interleukin-6 (IL-6), which operates through the Janus kinase 2 (JAK2) and signal transducer and activator of transcription 3 (STAT3) signaling cascade. The activation of the JAK2/STAT3 signaling in immature myeloid cells results in their conversion to myeloid-derived suppressor cells (MDSCs). The MDSCs then release a variety of their own modulatory factors that suppress the actions of cytotoxic T cells and natural killer cells, thereby limiting the immune response against the PDAC tumor [50]. Another protein released by activated PSCs is galectin-1, which is a part of the β-galactoside-binding family. Galectin-1 is a contributor to tumor invasion and metastasis [51]. This concept was further evaluated in a study done by Tang et al. that examined the role of galectin-1 in PDAC. When co-culturing CD3^+^ T cells with activated PSCs overexpressing galectin-1, the authors found that this led to significant apoptosis in the CD3^+^ T cells. The authors also found that galectin-1-overexpressing PSCs shifted the Th1/Th2 cytokine balance towards Th2 cytokine release, which facilitates immune cell evasion [52,53]. Furthermore, secretion of C-X-C Motif Chemokine Ligand 12 (CXC12) by activated PSCs assists in sequestering CD8^+^ T cells in the stroma distant from the tumor. The isolation of CD8^+^ T cells in this distant compartment significantly reduces infiltration into the tumor, thereby establishing the immunosuppressive environment [54]. 

Interleukin-10 (IL-10) and TGF-β are potent immunosuppressive cytokines that are released into the TME by PDAC cells and immune cells during tumorigenesis [55,56]. These cytokines recruit regulatory T cells, which in turn also release IL-10 and TGF-β, inhibiting effector T cells and maintaining immunosuppression [57,58,59]. The presence of IL-10 and TGF-β in the PDAC TME also shift the Th1/Th2 cytokine balance towards Th2 cytokine release, thereby further enhancing the immunosuppressive TME [59,60].

Intra-pancreatic γδ T cells indirectly support PDAC pathogenesis by inhibition of αβ T cells using checkpoint receptor ligation [61]. Seifert et al. found that γδ T cells interacted with PSCs and stimulated their production of IL-6, which leads to increasing amounts of ECM deposition in the PDAC stroma. Therefore, the interaction of γδ T cells with PSCs contributes to immunosuppression by fortifying the fibrotic barrier environment [62]. 

## 4. Strategies for Targeting Fibrosis in PDAC

Given the significant role for the ECM in pancreatic cancer progression, several different strategies have been investigated that target the fibrotic TME. Approaches currently under evaluation seek to either reduce stromal ECM deposition to improve the delivery of cytotoxic agents, or target ECM proteins for direct delivery of therapeutics to the tumor, thereby limiting off-target effects. 

Delivery of cytotoxic agents such as gemcitabine has been improved with the use of nab-paclitaxel [63,64]. Nab-paclitaxel/gemcitabine (AG) penetration was further improved in a phase 2 trial using pegvorhyaluronidase alfa (PEGPH20), but no substantial improvement was seen in a recent phase 3 clinical trial [65]. Treatment guidelines for borderline-resectable and locally advanced pancreatic cancers have recently shifted towards neoadjuvant therapy [66] and the use of combination therapies such as FOLFIRINOX [67,68]. Targeted delivery of FOLFIRINOX was improved using iontophoretic delivery [69]. Directed chemotherapy measures tend to have less associated side effects than systemic chemotherapy. Thus, these localized treatment strategies as a method to overcome ECM deposition warrant further investigation. 

The literature describes a variety of strategies used to target CAFs in PDAC. In general, these methods include conversion of CAFs to their quiescent phenotypes, inhibition of CAF signaling cascades, depletion of CAFs, use of CAFs as a cellular vehicle for cyto-toxic agents, and targeting of CAF-derived ECM proteins [70,71,72]. Some previous studies targeting CAFs have unfortunately led to a more aggressive tumor [73] and/or severe side effects [74]. However, with the discovery of CAF heterogeneity, specific targeting of CAFs could lead to improved therapeutic benefit [75,76]. Regarding clinical trials, several ongoing PDAC trials are investigating therapeutics that disrupt CAF signaling or reprogram CAFs to quiescence (Table 1). These clinical trials should be closely followed to determine if these stromal interventions increase chemotherapy efficacy.

Activated PSCs have a prominent role in PDAC ECM deposition. As a result, several investigations have been performed to elucidate therapeutic strategies against PSCs, hypothesizing that inhibition of these fibroblasts would lead to reduced fibrosis and, therefore, enhanced cytotoxic efficacy. For example, epidermal growth factor receptor (EGFR) can activate PSCs, and it was found that inhibition of EGFR reduced fibrosis [77]. PSCs can also be activated to increase desmoplasia by the Sonic Hedgehog (SHH) protein as part of the hedgehog pathway [78]. Studies impeding SHH signaling have led to mixed results [36,79]. Targeting the renin-angiotensin system, which has been demonstrated to activate PSCs [80], using olmesartan, an angiotensin II type-1 receptor blocker, decreased collagen deposition of PSCs, in vitro [81]. More recent PSC-targeting strategies include the use of phytochemicals such as curcumin, which can hinder the gene expression of type I and III collagen, thereby decreasing fibrotic production [82]. Future PSC inhibition studies will likely make use of more directed treatment strategies such as nanotechnology [83]. These strategies aim to reduce PSC activation to decrease ECM deposition and improve efficacy of treatments that are limited by substantial fibrosis in the TME.

Another strategy may resurrect the century old concept of therapeutic infection, as introduced by the sarcoma surgeon Dr. William Coley with “Coley’s toxins” [84], using the natural properties of infectious agents such as bacteria to target the TME and modulate anti-cancer immune responses. Group A Streptococcus Streptococcal collagen-like protein 1 (Scl1), is a major GAS adhesin, which exhibits selective binding to ECM proteins [85]. Scl1 binds to tumor-associated isoforms of cellular fibronectin (cFn) containing type IIII repeats, extra domain A and/or B (EDA/EDB/cFn) also known as oncofetal Fn [86,87,88]. Binding to EDA and EDB is mediated through conserved structural determinants present within the Scl1 globular V domain and facilitates GAS adherence and biofilm formation in the host [89,90,91]. In vitro, Scl1 mediates biofilm formation on matrices deposited by cancer-associated fibroblasts (CAFs) and osteosarcoma (Saos-2) cells containing EDA/EDB/cFn isoforms [87,89]. Importantly, oncofetal cFn is expressed in many cancers [92], including pancreatic tumors [93], suggesting a potential for bacterial targeting of tumors by Scl1 after injection [94]. Indeed, EDB expression in pancreatic tumors has been leveraged to develop imaging probes for EDB fibronectin to visualize pancreatic tumors [95,96]. 

While CAF-targeting and therapeutic infection are promising areas of continued research, studies thus far have not yet fully revealed definitive clinical benefit. Therefore, it is important to simultaneously explore other therapeutic options for a highly aggressive malignancy such as PDAC. Another exciting field of treatment focuses on targeting immune cells, especially neutrophils and macrophages, due to their significant pro-fibrotic effects in the PDAC TME. Successful targeting of these immune cells has the potential to mitigate both immunosuppression and fibrosis.

## 5. Immune Characterization of the TME and Impact on the ECM

As mentioned previously, the TME harbors a heterogenous population of immune cells such as macrophages, neutrophils, dendritic cells, natural killer cells, effector T lymphocytes, regulatory T lymphocytes, MDSCs, and B lymphocytes [97]. In general, immune cells modulate the TME through direct interactions with the tumor or indirectly by releasing a variety of chemical mediators. These cellular communications can both facilitate and hinder the effectiveness of therapeutics in the TME [98]. Given the significant fibrotic barrier in the PDAC TME that obstructs therapeutic delivery, it is important to explore the contribution of immune cells to fibrosis. Specifically, neutrophils and macrophages have been implicated for their role in ECM deposition. Neutrophils and macrophages in physiologic settings contribute to the natural wound healing process in injured tissue without fibrosis. However, pathological disruptions in this homeostatic mechanism can result in a fibrotic phenotype [99]. Therefore, in this section we will examine both key immune cells to elucidate their mechanisms of ECM modulation. 

### 5.1. Neutrophils

Neutrophils, also known as polymorphonuclear leukocytes, are the most common circulating leukocyte and play a key role in microbial defense [100,101]. Classic effector immune responses of neutrophils include phagocytosis and secretion of hydrolytic enzymes, granule-derived myeloperoxidase, and antimicrobial proteins/peptides. Additionally, neutrophils further participate in the immune response by releasing lipid mediators, cytokines, chemokines, and extracellular vesicles [102]. 

Recruitment signals into the TME for neutrophils include the ligands that bind to CXCR2, such as CXCL1 and CXCL2 [103]. It is also likely that tumor-derived GM-CSF recruits neutrophils into the TME, as this mechanism has been implicated in other cancers including gastric adenocarcinoma [104]. Like macrophages, neutrophils in the TME are capable of polarizing into different phenotypes: N1 and N2. Although the N1/N2 terminology facilitates discussion of these phenotypes, these cells function on a spectrum, therefore, our preference is to describe them as N1-like and N2-like. Conversion into either phenotype designates the neutrophil as a tumor-associated neutrophil (TAN). The N1-like phenotype is considered anti-tumorigenic as it releases reactive oxygen species (ROS), Fas, intercellular adhesion molecule (ICAM)-1, and tumor necrosis factor (TNF-α). These products are cytotoxic towards the tumor and hinder immunosuppression of the TME. The N2-like phenotype appears to promote tumorigenesis by remodeling the ECM and supporting angiogenesis of the tumor. This is accomplished through secretion of arginase, MMP-9, VEGF, and a variety of chemokines [105,106]. ECM remodeling by MMP-9 facilitates release and subsequent activation of VEGF from the ECM, thereby increasing vascularization of the tumor [107]. 

Neutrophils can also neutralize bacteria and other pathogens through formation of neutrophil extracellular traps (NETs) [108]. In this process, neutrophils release decondensed DNA, histones, high mobility group box 1 protein (HMGB1), ROS, and granules that ensnare and kill bacteria [108,109]. Typically, the expulsion of intracellular contents is a slow process that occurs as the neutrophil is dying. However, an alternative NET mechanism can occur that is independent of cell death and results in expedited degranulation [110,111]. In the unstimulated neutrophil, the DNA is tightly wrapped around histones and stored as heterochromatin. Upon exposure to the pathogen, the heterochromatin is decondensed by peptidyl arginine deiminase 4 (PAD4), which catalyzes the citrullination of histones [109,112]. Decondensation of histones is also facilitated from the interaction between histones and neutrophil elastase (NE) after NE translocation into the cell nucleus [110]. 

Although NETs are beneficial for protection against microbes, recent studies have shown that they contribute to pathogenesis of sterile inflammatory diseases including PDAC [113]. Neutrophils from PDAC are primed for NET formation and NETs are increased in both the circulation and TME during PDAC progression [114,115,116]. NETs promote the pathogenesis through multiple mechanisms including stimulating primary pancreatic tumor growth [114], driving cancer-associated hypercoagulability [117,118,119,120], promoting formation of metastatic disease [121,122,123], and supporting immunosuppression [124,125]. Several studies have also implicated NETs in activating PSCs to modulate the TME. PSCs transform into the activated state upon binding extracellular DNA [126], which is a predominant component of NETs. Indeed, interactions between the receptor for advanced glycation end products (RAGE) on quiescent PSCs and the DNA released from neutrophils during NETosis result in activation of PSCs [114]. As mentioned previously, activated PSCs heavily contribute to the fibrotic stroma of PDAC through deposition of ECM proteins, providing a mechanism through which neutrophils and NETs promote the fibrotic TME. 

### 5.2. Factors Promoting Neutrophil Extracellular Traps in PDAC

Given the expansive role for NETs in pancreatic pathogenesis, identifying the signals that trigger NETosis is critical to targeting this cancer-promoting phenomenon. Numerous potential targets have been identified. NET formation is dependent on RAGE, as mice with RAGE knockout resulted in significantly decreased extracellular DNA [115]. A recent study done by Zhang et al. explored the role of IL-17 in PDAC tumorigenesis and immunosuppression. Using an in vitro NET formation assay, the authors discovered that when KPC cells from spontaneous PDAC mice preconditioned with IL-17 were used as conditioned media for neutrophils, NET formation was significantly higher than control neutrophils exposed to IL-17 alone. The authors also found a similar significant result for TNFα [124]. Thus, it is likely that IL-17 and TNFα are crucial factors involved in the recruitment of TANs and NETs. 

A recent study reported that amyloid fibrils, insoluble fibers resistant to degradation, can trigger neutrophils into NET activation [127]. To determine if amyloid fibrils contributed to NET activation in the pancreatic tumor microenvironment, Munir et al. used mass spectrometry to investigate the presence of amyloid proteins. They found that the Amyloid β A4 protein (APP) was highly expressed in CAFs. Interestingly, they found that *APP* mRNA was also found in PSCs, though to a lesser extent. The authors showed that CAFs induce NET formation, but by inhibiting secretion of APP, found that CAFs were unable to stimulate NETs. Moreover, through blocking the potential APP receptor, CD11b, the authors noted that neutrophils were no longer stimulated into NETosis [128], implicating several potential targets for NET formation in PDAC. 

Research into the stimuli for NETs in PDAC is relatively new, so it is also important to explore how NETs are promoted in other forms of cancer. A recent study done by Li et al. examined the function of Sciellin (SCEL), a precursor to the cornified envelope, which is a protective barrier in the upper epidermis [129], in gallbladder cancer progression. Using a co-culture experiment, the authors found that SCEL induced expression of NETs and citrullinated-histone 3, which is a critical marker of NET formation [130]. Similar to gallbladder cancer, SCEL is markedly elevated in pancreatic cancer [131]. Therefore, it would be integral for future studies to examine if SCEL participates in neutrophil/NET recruitment in PDAC. 

### 5.3. Therapeutic Strategies for Targeting Neutrophils and NETs

**Figure 3:**
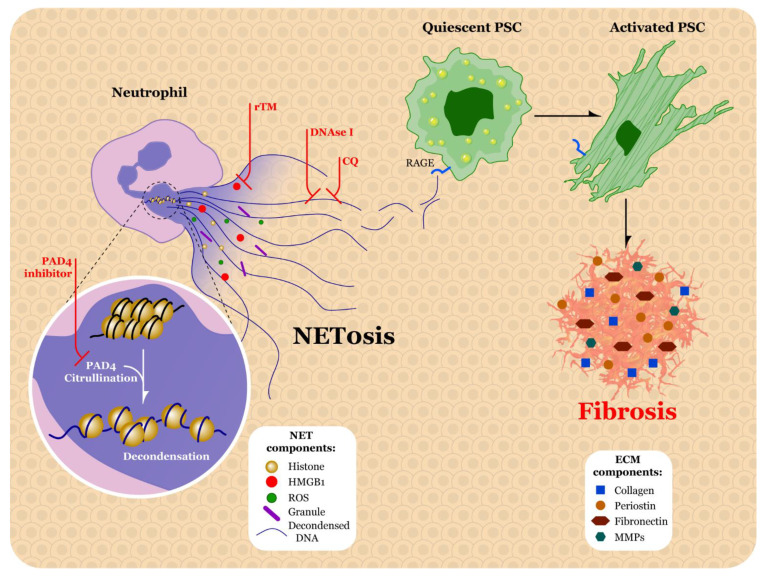
Proposed neutrophil extracellular trap (NET) targeting strategies. Peptidyl arginine deiminase 4 (PAD4) inhibitors block PAD4-mediated citrullination of histones, thereby preventing heterochromatin decondensation and subsequent NET release. Recombinant thrombomodulin (rTM) can inhibit high mobility group box 1 protein (HMGB1), reducing capture and migration of tumor cells by NETs. DNAse I and chloroquine (CQ) degrade and decrease secretion of decondensed DNA, respectively, thereby reducing interactions of decondensed DNA with the receptor for advanced glycation end products (RAGE), preventing pancreatic stellate cell (PSC) activation and subsequent extracellular matrix (ECM) deposition. ROS, reactive oxygen species; MMPs, matrix metalloproteinases.

Research over the past few years has investigated potential therapeutic strategies for NETs (Figure 3). In addition to promoting inflammation, NETs can also lead to coagulation as the expelled intracellular contents create a scaffold for thrombus formation [132]. A recent study done by Kajioka et al. found that NETs can capture PDAC cells and influence their migration and invasion capabilities. The authors also explored the possibility of targeting NETs in pancreatic cancer cells using recombinant thrombomodulin (rTM), a type of endothelial cell surface protein. The authors found that, when treating pancreatic cancer cells with rTM, HMGB1 released from NETs was degraded. As a result, the capture/migration of PDAC tumor cells controlled by NETs was inhibited thereby reducing metastasis to the liver [121]. TM has also been tested against NETs in situations other than pancreatic cancer. For example, it was determined that rTM reduced histone-induced NET release via citrullinated histone 3 staining in kidney sections of rTM-treated rats [133]. Another study examined the impact of rTM treatment on NETs in septic shock rat models as this condition leads to intravascular coagulation. The authors discovered that rTM treatment reduced systemic NETs in septic shock rat models compared to control, as determined by examining levels of citrullinated histone H3 and DNA, or NE and DNA [134]. The reduction in NETs seen in these studies show promising results for rTM, and this treatment strategy should continue to be thoroughly evaluated in additional pancreatic cancer studies. 

A second therapeutic strategy for NETs is targeting the DNA that is expelled from neutrophils during this process. Deoxyribonuclease I (DNAse I) treatment of murine acute lung injury models induces degradation of NETs structure [135]. In another study related to lung injury, when methicillin-resistant *Staphylococcus aureus* (MRSA)-infected mice were treated with DNase I, it was found that neutrophil elastase-DNA (NE-DNA) ELISA measurements were reduced in bronchoalveolar lavage blood [136]. As of this writing, the severe acute respiratory syndrome coronavirus 2/coronavirus disease 2019 (SARS-CoV-2/COVID-19) pandemic is still ongoing. SARS-CoV-2 has been associated with excessive NETs and coagulation [137]. A study done by Park et al. treated severe SARS-CoV-2 patients with either free DNase I or DNase-I-coated melanin-like nanospheres (DNase-I pMNSs). With both treatments, the authors noted significant decreases in extracellular DNA, NET percentage, myeloperoxidase activity, and NE, along with a significant increase in relative plasma DNase activity. Interestingly, treatment with DNase-I pMNSs was more effective than free DNase I at reducing cytokine secretion from neutrophils such as NF-κβ and TNF-α [138]. As a result of these beneficial effects, it is worth exploring the use of DNase-I nanotechnology in the setting of PDAC NETs. Although many DNase I studies have focused on lung injury, a study done by Xia et al. examined the impact of DNase I in the setting of colorectal cancer metastasis to the liver. Due to the short biological half-life of DNase I, the investigators used adeno-associated virus (AAV) as the vector for long-term expression of the enzyme in the liver. The authors found decreased levels of citrullinated histone H3 and NETs in the tumors of mice treated with AAV-DNase I, as compared to the tumors in mice that were treated with AAV-null [139]. 

Another therapeutic approach to NET inhibition involves the use of chloroquine (CQ). In a study examining the potential effect of NETs on high density lipoprotein (HDL) in systemic lupus erythematosus (SLE), Smith et al. found that CQ hindered NET formation in both the control neutrophils and in a type of peripheral blood lupus neutrophils called low-density granulocytes [140]. Treatment with chloroquine resulted in a decrease in serum DNA in the Kras pancreatic cancer mouse model. Moreover, of 15 PDAC patients who were treated with neoadjuvant gemcitabine plus hydroxychloroquine, 12 patients had a significant reduction in circulating DNA levels [115]. Inhibition of NETs by CQ also reverses the hypercoagulable state seen in PDAC [119]. It is also important to note that PAD4 deficiency has been shown to reduce NET formation, and, therefore, PAD4 inhibitor treatments should also be thoroughly investigated as a potential therapeutic target [119,141]. 

There are a few clinical trials relevant to the treatment of NETs, although they are outside the context of PDAC. Table 2 delineates current NET targeting strategies in clinical trials. One clinical trial (NCT03250689) examined the effect of Danirixin, a selective CXCR2 antagonist, on NETs in chronic obstructive pulmonary disease (COPD) patients. The study was eventually terminated due to changes in the benefit risk profile of Danirixin that was determined in another clinical trial (NCT03034967). A clinical trial at McGill University Health Center, DISCONNECT-1 (NCT04409925) is currently recruiting to evaluate the safety of inhaled rhDNase I and its impact on NETs in severe SARS-CoV-2 patients. Another clinical trial (NCT03368092) at University Hospital in Strasbourg, France, is also evaluating the effect of inhaled rhDNase I on NET-induced lung injury.

### 5.4. Tumor Associated Macrophages

Macrophages are a group of immune cells that possess heterogenous function and serve as the first line of immune protection in nearly every tissue [142]. An over-simplified view of macrophage differentiation is that macrophages undergo polarization into different phenotypes depending on the cytokine exposure. The classical activation pathway, in the presence of Th1-derived cytokines such as IFN-γ, colony stimulating factor 2 (CSF2), or toll-like receptor (TLR) activation, gives rise to the M1-like macrophage phenotype considered more protective against cancer cells. The alternatively activated pathway, in the presence of Th2-derived cytokines such as IL-4, IL-10, IL-13, TGF-β, prostaglandin E2, or colony stimulating factor 1 (CSF1), gives rise to the M2-like macrophage phenotype, which typically facilitates tumor progression [143,144]. However, it is now understood that macrophage polarization extends beyond the dichotomy of M1/M2 phenotypes and is better defined as a spectrum [145,146]. Tumor-associated macrophages (TAMs) are considered to have an M1-like phenotype during the early process of tumorigenesis, and then eventually switch to an M2-like phenotype [147]. In the TME, TAMs contribute to PDAC pathogenesis through their promotion of inflammation, tumor angiogenesis, metastasis, immune evasion, and ECM modulation [148]. In this section, we will focus on the various mechanisms by which TAMs alter the ECM. 

### 5.5. Effect of TAMs on ECM

There are several investigations in the literature that implicate TAMs for their role in enhancing the ECM deposition in PDAC. Co-culturing quiescent pancreatic stellate cells with macrophage cell lines in the presence of Heparin-binding EGF (HB-EGF) activates stellate cells and promotes α-SMA expression [149,150]. Activation of these pancreatic stellate cells likely lead to increased deposition of ECM proteins in the tumor stroma. When comparing human pancreatic tissue samples possessing both PDAC lesions and adjacent unaffected tissue, Zhu et al. found a positive correlation between amount of tissue fibrosis and number of macrophages. This group also found, through analysis of gene ontology, that embryonically-derived macrophages expressed higher levels of ECM remodeling genes as compared to monocyte-derived macrophages. For example, qPCR demonstrated higher expression levels of the ECM-producing enzymes hyaluronan synthases 2 and 3 [151]. Activated M2-like macrophages participate in ECM remodeling by secreting MMPs, which exert digestive effects on the ECM [152]. A recent study done by Tekin et al. analyzed mRNA expression of various proteases by quiescent macrophages in the TME. The authors found that MMP9 was significantly produced and led to cleavage of protease-activated receptor-1 (PAR1), a G protein-coupled receptor linked to tumor growth [153]. Because MMPs participate in ECM remodeling, they play a significant role in the levels of fibrosis in the TME. 

Tissue resident macrophages, the predominate macrophage subsets in the pancreatic TME, express the prolactin receptor [154] and prolactin has been reported to contribute to the fibrosis of the TME. One of the downstream effectors of prolactin receptor signaling is focal adhesion kinase 1 (FAK1). Treatment with a FAK1 inhibitor significantly decreases fibrosis in transgenic models of murine pancreatic cancer. Therefore, activation of tissue-resident macrophages by prolactin could regulate collagen deposition in the TME.

Incubation of murine fibroblasts with macrophages containing the lipid kinase PI3Ky increased collagen mRNA in those fibroblasts as compared to murine fibroblasts incubated with PI3Ky-deficient macrophages. Additionally, it was found that pancreata from both PI3Ky-deficient KPC mice and KPC mice treated with a PI3Ky inhibitor displayed significantly less fibrosis as compared to controls. Reduced collagen protein and gene expression was also observed in orthotopic LMP tumors treated with a PI3Ky inhibitor [155]. As these PI3Ky-macrophages are present in PDAC, their pro-fibrotic effects and potential as therapeutic targets necessitate further investigation. 

Macrophages are also involved in establishing a pre-metastatic niche that promotes PDAC metastasis to the liver, suggesting a role for macrophages modulating fibrosis in the TME. In a study done by Nielsen et al., it was determined that, after exposure to a variety of cancer cell derived factors, M2-like macrophages and metastasis-associated macrophages release granulin. Not only does the secretion of granulin itself likely contribute to the fibrotic stroma, it also activates resident hepatic stellate cells promoting their differentiation into myofibroblasts. These myofibroblasts then release a number of proteins related to ECM remodeling. In particular, the myofibroblasts release high levels of periostin, which contribute to the fibrotic stroma in the TME and facilitate pancreatic tumor growth and invasion into the liver [156]. 

In addition to PDAC, macrophages are known to contribute to fibrosis in other disease models. For example, it has been recently shown that macrophages expressing the AP-1 transcription factor Fra-2 contributes to the ECM deposition in idiopathic pulmonary fibrosis by releasing type VI collagen. It is unclear if there are Fra-2-expressing macrophages present in the PDAC TME. Therefore, an interesting future investigation would be to search for the existence of these specific macrophages in the PDAC TME as it would be another factor leading to the desmoplastic reaction [157]. 

Tunica Interna endothelial cell kinase (Tie2)-expressing macrophages (TEMs) are a distinct subtype of macrophages considered to be highly pro-angiogenic and immunosuppressive in the TME [158,159,160]. Tie-2 expressing macrophages have been associated with poor survival in gastric cancer patients [161] and in PDAC patients when M2-like TAMs are also present [162]. Tie2 is a receptor tyrosine kinase that binds to angiopoietin 1 (ANG-1) and angiopoietin 2 (ANG-2). In circulation, TEMs highly express the pro-angiogenic genes *MMP-9*, *VEGFA, COX-2*, and *WNT5A.* In the TME, ANG-2 is secreted by endothelial cells and sometimes tumor cells. ANG-2 levels are typically higher than ANG-1 in the TME. Binding of ANG-2 in the TME leads to upregulation of two other pro-angiogenic genes cathepsin-B (*CTSB)* and thymidine phosphorylase (*TP)* as well as the highly immunosuppressive *IL-10* [163]. To our knowledge, there are currently no studies available on TEM-mediated fibrosis in the TME. Given the substantial modulatory functions of TEMs on TME angiogenesis and immunosuppression, an analysis of their potential role in PDAC fibrosis would further contribute to their recent growing importance as a therapeutic target. 

### 5.6. Recruitment of Macrophages

Various chemokines and cytokines released by the tumor promote macrophage recruitment into the TME. IL-4, IL-10, IL-13, IL-34, TGF-β, and complement component C5a all have been implicated in this macrophage recruitment. Colony-stimulating factor (CSF)-1 leads to myeloid progenitor differentiation into monocytes and macrophages. Moreover, it has been demonstrated that CSF-1 is involved in generating the M2-like phenotype of macrophages [164,165,166]. As pancreatic tumorigenesis proceeds, C-C motif chemokine ligand 2 (CCL2) is released by the tumor cells, leading to substantial attraction of circulating monocytes to the TME. Further, the imbalanced release of various chemical mediators such as CCL5, CCL7, CXCL8, CXCL12, and VEGF also serve as chemoattractants for macrophages and facilitate conversion into the M2-like phenotype [152]. Additionally, a recent study done by Tekin et al. demonstrates a positive correlation between number of macrophages in the TME and PAR1 expression in pancreatic tumor tissues [153]. 

Pancreatic acinar cells with *KRAS* mutation express intercellular adhesion molecule-1 (ICAM-1), which can recruit macrophages to the TME. The infiltration of macrophages facilitates the conversion of acinar to ductal phenotype, which is an integral early component of pancreas carcinogenesis [149]. Two subsets of macrophages are present within the pancreatic inter-acinar stroma. One population is derived from primitive hematopoiesis, whereas the other population is derived from definitive hematopoiesis and substitutions with circulating myeloid cells [151,167].

### 5.7. Strategies to Target Macrophages

**Figure 4:**
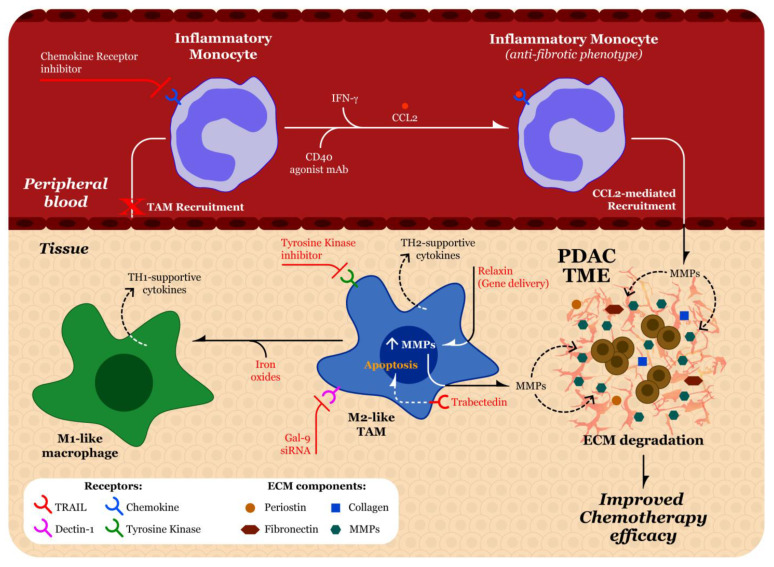
Potential tumor associated macrophage (TAM) targeting strategies. Strategies have been developed that target macrophages in the peripheral blood (top panel) and the PDAC TME (bottom panel). Inhibition of the chemokine receptor may decrease recruitment of inflammatory monocytes into the tissue, where they can polarize into M2-like TAMs. Treatment with a CD40 agonist monoclonal antibody (mAb) can increase systemic levels of interferon-γ (IFN-γ), thereby polarizing the inflammatory monocyte into an anti-fibrotic phenotype. The binding of CCL2 then recruits the inflammatory monocyte into the tissue, where it releases various matrix metalloproteinases (MMPs) that can degrade the abundant extracellular matrix (ECM) of the pancreatic ductal adenocarcinoma tumor microenvironment (PDAC TME), improving chemotherapy efficacy. In general, M1-like macrophages tend to release TH1-supportive cytokines, which are considered more protective against cancer cells. M2-like TAMs usually release TH2-supportive cytokines, which tend to support cancer progression. The literature describes a variety of methods (noted in red text) to target M2-like TAMs. Inhibition of the tyrosine kinase receptor may reduce survival and proliferation of M2-like TAMs. Both iron oxides and targeting of the dectin-1 receptor by galectin-9 small interfering RNA (siRNA) can repolarize M2-like TAMs into the M1-like phenotype. Trabectedin operates through the TNF-related apoptosis-inducing ligand (TRAIL) receptor, thereby leading to apoptosis of the M2-like TAM or switching their secretion profile to that of an inflammatory phenotype. Gene delivery of relaxin (RLN) can upregulate MMP9 and MMP13 genes, thereby leading to increased release of MMPs into the PDAC TME that can degrade fibrosis and improve chemotherapy efficacy.

There are several different therapeutic strategies described in the literature that seek to reduce the impact of TAMs in the TME (Figure 4). In general, these therapeutic options target different properties of TAMs such as their survival, polarization, recruitment, phagocytosis, and angiogenesis [168]. Different pharmacological techniques for TAMs such as targeting chemokine-chemokine receptors and tyrosine kinases, as well as the use of bisphosphonates and nanotechnology have been evaluated [169]. Table 3 lists the clinical trials of therapeutics being tested against TAMs in pancreatic cancer. In this section, we will discuss some of the more recent and novel pharmacological approaches to attenuate the influences of TAMs on ECM production. 

Targeting chemokine-chemokine signaling represents a promising strategy to limit macrophage infiltration. Targeting the CCL2/CCR2 axis in recruitment of CCR2^+^ inflammatory monocytes to the PDAC TME, where they can differentiate into macrophages, is one strategy that has been examined. The small molecule CCR2 inhibitor, PF-04136309, decreases levels of circulating inflammatory monocytes in tumor bearing mice, effectively blocking TAM recruitment to the PDAC TME [170]. The safety and efficacy of PF-04136309 along with FOLFIRINOX on TAMs in PDAC was tested in a recent phase Ib clinical trial (NCT01413022). Of 47 enrolled patients, 39 received the combination therapy, whereas eight patients received FOLFIRINOX alone. Using flow cytometry on six post combination therapy treatment tumor biopsies, the authors found a mean reduction in TAMs from 9.0% to 2.4%. Further, they found a significant decrease in peripheral blood CCR2^+^ monocytes with the combination therapy as compared to FOLFIRINOX alone, which indicates blockage of TAM recruitment by the PDAC TME [171]. 

Another strategy in treating TAMs involves the targeting of tyrosine kinases. As mentioned previously, CSF-1 is a cytokine involved in the polarization of macrophages into a tumor-supportive phenotype. In general, efforts to target the CSF-1/CSF-1R interaction are CSF-1/CSF-1R antibodies and CSF-1R kinase inhibitors. A randomized phase 2b clinical trial (NCT03336216) tested the efficacy of cabiralizumab, an antibody that blocks CSF-1R, in combination with nivolumab, with or without chemotherapy, in patients with advanced pancreatic cancer. Unfortunately, the clinical trial sponsor reported that the combination therapy with and without chemotherapy was not beneficial compared to standard chemotherapy [172]. A recent phase 1b trial (NCT02713529) was completed that tested the safety and efficacy of AMG 820, an anti-CSF1R monoclonal antibody, in combination with anti-PD-1 antibody pembrolizumab in adults with advanced pancreatic cancer, colorectal cancer (CRC), or non-small cell lung cancer (NSCLC). Although AMG 820 plus pembrolizumab was shown to have an adequate safety profile, none of the pancreatic cancer patients met the pre-determined threshold for efficacy. There were two CRC patients and one NSCLC patient who achieved a response of immune-related partial response [173]. Blocking tyrosine kinases is also being evaluated for several other types of gastrointestinal cancers, hopefully leading to more progress in this therapeutic strategy. 

A third strategy recently evaluated for targeting macrophage polarization is the use of nanotechnology. Several types of nanoparticles that are used to target TAMs have been evaluated [174]. Iron oxides have been demonstrated to switch the polarization of M2-like macrophages into the M1-like phenotype. Additionally, iron oxides increase ROS production and induce apoptosis in cancer cells [175]. In a study done by Zhao et al., the authors developed a tumor-derived antigenic microparticle (T-MP) that contained nano-iron oxide. The surface of the T-MP was tethered with adjuvant CPG oligodeoxynucleotides-loaded liposomes. Once this combination vaccine was delivered into the TME, it was determined that repolarization of M2-like macrophages to M1-like had occurred [176]. Interference with the galectin-9/dectin axis, which has been previously implicated in the conversion of macrophages to the M2-like phenotype, is another strategy targeting the polarization of macrophages in the PDAC TME. A nanoscale delivery system composed of bone marrow mesenchymal stem cell exosomes that were electropermeabilization-loaded with galectin-9 siRNA has been evaluated [177]. Additionally, these exosomes contained oxaliplatin to induce death in tumor cells. After co-delivery of the siRNA and oxaliplatin into orthotopic pancreatic tumor-bearing C57BL/6J mice, the authors found significant re-polarization of TAMs into the M1-like phenotype via flow cytometry and immunofluorescence staining of tumor sections, using CD206 as a marker for M2-like macrophages and CD 16/32 for the M1-like phenotype [177]. Given the many potential benefits of nanotechnology, such as increased stability and decreased side effects [178], they are certainly worth continued exploration for application in targeting TAMs in the PDAC TME. 

Macrophage polarization has also been targeted using trabectedin, an isoquinoline cytotoxic agent that was initially isolated from a Caribbean tunicate [179]. Trabectedin can operate through the monocyte specific TNF-related apoptosis-inducing ligand (TRAIL) receptors 1 and 2, resulting in the extrinsic apoptotic pathway through activation of caspase-8 [180]. A study using a patient-derived orthotopic mouse model of gemcitabine-resistant PDAC reported that treatment with trabectedin inhibited but did not regress PDAC tumor growth [181]. In a PDAC mouse model, it was shown that depletion of TAMs by trabectedin significantly increased infiltration of CD4 and CD8 T cells into the TME. Notably, in trabectedin treated mice, both infiltrating CD4 and CD8 T cells produced lower levels of the immunosuppressive cytokine IL-10. CD4 T cells also produced increased levels of IFN-γ. Lastly, it was demonstrated there was higher levels of TAM secretion of inflammatory mediators such as IL2, IL12, IL17, and TNFα, suggesting a switch to the inflammatory M1-like phenotype of TAMs [182]. It has not yet been explored whether this possible re-polarization by trabectedin could lead to reduced fibrosis in the PDAC TME. Trabectedin was approved in 2015 by the Food and Drug Administration for treatment of liposarcomas and leiomyosarcomas and, therefore, has not yet been extensively evaluated for use in PDAC [183]. A phase II clinical trial tested single agent trabectedin in patients with gemcitabine resistant metastatic PDAC. Unfortunately, the primary endpoint measure of progression-free survival at six months from treatment was not met [184]. An interesting future investigation should analyze the impact of trabectedin on PDAC fibrosis and determine if combination with other cytotoxic agents improves clinical outcomes. 

Some investigators have found success by reprogramming TAMs to deplete fibrosis. Treatment with an agonist CD40 monoclonal antibody increased the systemic release of IFN-γ, leading to polarization of CCR2^+^ monocytes into an anti-fibrotic phenotype. These inflammatory monocytes are then recruited into the PDAC TME via CCL2 release. Once in the TME, monocytes differentiate to inflammatory macrophages that are able to release various MMPs that deplete ECM proteins such as fibronectin and type I collagen, thereby reducing fibrosis and increasing the efficacy of cytotoxic agents in the PDAC TME [185]. Previous studies have shown the beneficial effect of the anti-fibrotic hormone relaxin (RLN) in reducing fibrosis in PDAC and liver metastasis from various cancers [186,187]. A study done by Zhou et al. found that more than 70% of cells in both macrophage and fibroblast populations expressed the relaxin family peptide receptor type 1 (RXFP1). After *RLN* gene delivery, the authors observed significant increases *MMP9* and *MMP13* mRNAs in the PDAC TME compared to the PBS-treated group, thereby leading to ECM degradation [188]. 

Regarding treatment strategies for TEMs, disruption of the ANG-2/Tie2 signaling pathway in vivo has been shown to inhibit tumor growth and reduce tumor microvasculature using monoclonal antibodies [189,190] and peptides [191]. A recent study using rebastinib, a selective inhibitor of Tie2, decreased both Tie2-expressing macrophage infiltration and TME vasculature density in a mouse model of mammary cancer, but reduced only Tie2-expressing macrophage infiltration in a pancreatic neuroendocrine tumor model [192]. Rebastinib is currently being evaluated in clinical trials in combination with chemotherapy for treatment of metastatic breast cancer (NCT02824575) and other advanced solid tumors (NCT03717415 and NCT03601897). To our knowledge, there have been no studies or clinical trials targeting Tie2-expressing macrophages in the context of PDAC. 

### 5.8. Beyond Neutrophils and Macrophages

Neutrophils and macrophages are the most extensively investigated immune cell types regarding fibrotic production. Generally, the involvement of immune cells appears to contribute to fibrosis in many disease contexts. Some immune cells, such as regulatory T cells (Tregs) and natural killer T (NKT) cells, have conflicting roles in fibrosis [193]. For example, in the TME, factors released from CAFs such as TGF-β cause Tregs to release their own TGF-β, which influences the conversion of quiescent fibroblasts into CAFs, likely promoting ECM deposition [194]. Although, in a study examining human immunodeficiency virus type 1 (HIV-1) infection in a humanized mouse model, the presence of Tregs mitigates liver fibrosis [195]. NKT cells have been shown to reduce collagen in the liver by selectively removing hepatic stellate cells after treatment with IL-30 [196]. In contrast, NKT cells have also been implicated in fibrosis production following liver injury through a CXCR6-dependent mechanism [197].

Dendritic cells release MMP9, which can have modulatory effects on the ECM, but more studies are required to clarify their relationship with fibrogenesis [198]. There is evidence that both T helper 2 and T helper 17 cells activate hepatic stellate cells, which in turn secrete collagen [199]. T helper 17 cells can release IL-17, which can promote hepatic stellate cell expression of collagen I and influence their conversion into fibrogenic myofibroblasts [200]. As mentioned earlier, γδ T cells have been demonstrated to contribute to both the immunosuppressive and fibrotic TME in PDAC [62]. Overall, crosstalk between various immune cells and CAFs are evident [201], but several more studies are needed to investigate the potential pro-fibrotic or anti-fibrotic roles of the various immune cells in PDAC specifically. 

## 6. Conclusions and Future Perspectives

The desmoplastic reaction heavily contributes to the poor prognosis of PDAC. The overabundance of ECM proteins establishes a fibrotic stroma and TME that are highly refractory to cytotoxic chemotherapy, immunotherapy, and radiotherapy. Targeting the stroma directly in pre-clinical studies has unfortunately led to inconsistent results and in some instances, a more aggressive disease [202]. Thus, various other therapeutic options such as targeting immune cell modulation of the ECM should be explored. Although it has been known for some time that NETs can contribute to various pathologies, their effect on PSC activation is a relatively new discovery. Thus, the laboratory investigations that target NETs in PDAC models are also quite new and limited in number yet promising. To our knowledge, there are currently no active clinical trials targeting NETs in the context of PDAC. With regard to TAMs in PDAC, several more laboratory studies and clinical trials of therapeutic strategies have been published. Further pre-clinical studies using NET-targeting therapies in combination with neoadjuvant and/or adjuvant cytotoxic agents is warranted. This strategy is already being evaluated for TAMs in clinical trials. Targeting both NETs and TAMs could deplete some of the fibrosis surrounding the tumor, thereby enabling better penetration of cytotoxic agents into the TME. While current strategies have focused on either macrophage or neutrophil targeting, limited efforts have been made to target both immune cells [103], which may be critical for efficacy. With the recent advancements in chemotherapy such as FOLFORINOX and innovations in more directed cytotoxic delivery, the addition of immune cell-targeting agents could be the extra boost needed to win the battle against this devastating disease. 

## Figures and Tables

**Table 1:** Clinical Trials Targeting Fibroblasts in PDAC.

Strategy	Therapeutic	Trial Phase	Trial Status	NCT Number
Disrupt CAF Signaling	Tocilizumab	1b/2	Recruiting	NCT03193190
	Tocilizumab	2	Recruiting	NCT02767557
	Tocilizumab	2	Active	NCT04258150
	Siltuximab	1,2	Recruiting	NCT04191421
	Canakinumab	1	Recruiting	NCT04581343
	Plerixafor	2	Recruiting	NCT04177810
	Plerixafor	1	Completed	NCT02179970
	BL-8040	2	Active	NCT02826486
Reprogramming to Quiescence	ATRA ^1^	1	Completed	NCT03307148
	ATRA ^1^	2	Not yet recruiting	NCT04241276
	Vitamin D3	3	Recruiting	NCT03472833
	Paricalcitrol	2	Completed	NCT03331562
	Paricalcitrol	1	Recruiting	NCT03519308
	Paricalcitrol	2	Recruiting	NCT04617067
	Paricalcitrol	1	Active	NCT03883919
	Paricalcitrol	2	Recruiting	NCT04524702

Source: Clinicaltrials.gov. ^1^ All-trans retinoic acid.

**Table 2:** Clinical Trials Targeting NETs in Various Diseases.

Therapeutic	Trial Phase	Trail Status	Context	Trial ID
rhDNAse I	1	Recruiting	Severe SARS CoV-2 ^1^	NCT04409925
	3	Recruiting	Moderate to Severe ARDS ^2^	NCT03368092
Danirixin	2	Terminated	COPD ^3^	NCT03250689
NucleoCapture Device	N/A	Recruiting	SA-AKI ^4^	NCT04749238

Source: Clinicaltrials.gov. ^1^ Severe Acute Respiratory Syndrome Coronavirus 2. ^2^ Acute Respiratory Distress Syndrome. ^3^ Chronic Obstructive Pulmonary Disease. ^4^ Sepsis-Associated Acute Kidney Injury.

**Table 3:** Clinical Trials Targeting Tumor-Associated Macrophages in Pancreatic Cancer.

Target	Therapeutic	Trial Phase	Trial Status	Additional Interventions	Trial ID
CSF1-R	IMC-CS4 (LY3022855)	1	Recruiting	Cyclophosphamide, GVAX, Pembrolizumab	NCT03153410
	Cabiralizumab (FPA008)	1a/1b	Completed	Nivolumab	NCT02526017
	Cabiralizumab (FPA008)	2	Completed	Nivolumab +/− Chemotherapy	NCT03336216
	Pexidartinib	1	Completed	Durvalumab	NCT02777710
CSF1	MCS110	1b/2	Completed	PDR001	NCT02807844
CCR2	PF-04136309	1	Completed	FOLFIRINOX	NCT01413022
	PF-04136309	1b	Completed	nab-paclitaxel and gemcitabine	NCT02732938
	CCX872-B	1	Active	FOLFIRINOX	NCT02345408
CXCR4	BL-8040	2b	Active	Pembrolizumab	NCT02907099

Source: Clinicaltrials.gov.

## Data Availability

The data presented in this study are openly available.
